# Auditory Perception Outcomes in Children with Deafness and Additional Disabilities 12 Months After Cochlear Implant Activation

**DOI:** 10.3390/audiolres15030047

**Published:** 2025-04-24

**Authors:** Celia Martínez-Pantanalli, Sofía Bravo-Torres

**Affiliations:** 1 Psychology of Communication and Change, Department of Basic Developmental and Educational Psychology, Faculty of Psychology, Autonomous University of Barcelona, 08193 Barcelona, Spain; 2 Speech Therapist, Otorhinolaryngology Service—Dr. Luis Calvo Mackenna Hospital, Santiago 13123, Chile; sbravo@calvomackenna.cl; 3 Escuela de Fonoaudiología, Faculty of Health, University of Santo Tomás, Santiago 8370003, Chile

**Keywords:** cochlear implantation, speech perception, deafness with additional disabilities, pediatric implantation

## Abstract

**Background/Objectives:** This study aimed to evaluate the progress in auditory speech perception in a group of children with cochlear implants and additional disabilities, whose implants were implanted at a public hospital in southern Chile between 2013 and 2019. This population has historically been excluded from research due to uncertainties regarding their outcomes. **Methods:** All pediatric patients who received cochlear implants between 2013 and 2019 were considered for inclusion. After obtaining informed consent, relevant data were collected from their medical records. A total of 18 children met the inclusion criteria. Data analysis was performed using Jamovi software. **Results:** The minimum age at cochlear implant activation was 2 years, and the maximum was 16.1 years. The median Category of Auditory Performance (CAP) score was 0 pre-implantation and increased to 2 to 12 months post-implantation. **Conclusions:** Cochlear implantation provides clear benefits for children with additional disabilities. Although gains in auditory perception may be limited in some cases, implantation enables access to the world of sound. Even when oral language development is not fully achieved, parents frequently report positive changes in their children’s interaction with their environment, suggesting an overall improvement in quality of life.

## 1. Introduction

Hearing loss, or deafness, is defined as the inability to perceive sounds within normal levels, especially at frequencies of 500, 1000, 2000, and 4000 Hz. According to the World Health Organization (WHO), its prevalence in newborns has remained stable in recent years [1]. However, the age of detection has decreased thanks to the implementation of neonatal hearing screening programs [2,3], which allow for early intervention through hearing aids or cochlear implants, leading to better outcomes in auditory perception and language development.

In some cases, hearing loss is accompanied by other developmental disabilities, such as autism spectrum disorder (ASD), cerebral palsy, global developmental delays, or genetic syndromes. It is estimated that up to 40% of children with hearing loss have associated conditions that may interfere with early detection and the implementation of effective auditory interventions. These comorbidities generate substantial clinical heterogeneity, complicating the evaluation of outcomes and limiting the generalizability of existing studies [4,5]. According to Núñez-Batalla et al. [5], among individuals with deafness and additional disabilities, 20% present with more than one associated condition. These multiple disabilities can significantly hinder adequate progress in language development due to their cumulative impact on communication abilities.

For many years, children with hearing loss and additional disabilities were excluded from cochlear implant programs due to uncertainties about their outcomes [6,7]. However, recent studies have shown that this intervention can produce significant benefits in auditory perception, quality of life, and social interaction, even if gains in oral language development are more modest [5,8,9]. In Chile, the gradual incorporation of cochlear implants into the public health system has expanded access to more diverse populations, including children with associated disabilities, provided that certain anatomical and functional criteria are met [10,11,12,13].

Despite these advances, there remains a lack of research specifically analyzing cochlear implantation outcomes in children with additional disabilities [6,8,14]. While it is recognized that language development in this population is often lower compared to peers with typical hearing, the extent to which an implant enables functional improvements in auditory perception is still unclear [8]. This lack of evidence limits clinical decision-making and family guidance [15,16]. Therefore, it is essential to study this specific group using objective and contextually relevant measures.

In this context, the present study focuses on auditory speech perception—a core component of language development and a key indicator of cochlear implant outcomes. This ability can be evaluated using a range of clinically validated tests. In Chile, where this study was conducted, assessment protocols typically include evaluations of suprasegmental perception, vowel and consonant recognition, and open-set sentence lists, among others [17]. These tests generate scores that are classified according to the Categories of Auditory Performance (CAPs), which provide insight into the listening skills acquired by individuals using hearing aids or cochlear implants. The CAP scale, developed by Archbold et al. [18], categorizes functional listening abilities along a hierarchical continuum—from the detection of environmental sounds to understanding speech over the telephone with a familiar interlocutor. Its simplicity and adaptability to children with limited verbal expression make it a particularly valuable tool for clinical follow-up and outcome assessments across diverse populations [5,19].

Beyond its clinical utility, the CAP scale is a user-friendly tool designed for straightforward application and interpretation, even by non-specialist professionals and caregivers. It facilitates consistent monitoring of auditory development over time and allows for the quantification of auditory receptive abilities in individuals with profound hearing impairment within clinical contexts [20,21]. The scale assesses auditory perception across multiple domains, including awareness, identification, and interpretation of auditory stimuli processed by the central nervous system. Table 1 outlines the eight hierarchical levels of the CAP scale, providing a structured overview of functional listening abilities.

This study aims to evaluate progress in auditory speech perception in a group of children with hearing loss and associated conditions, 12 months after cochlear implant activation. By focusing on a historically underrepresented population, this work seeks to provide empirical evidence to guide future interventions, inform inclusive public policy design, and expand the understanding of cochlear implant benefits beyond spoken language development.

## 2. Materials and Methods

### 2.1. Participants and Study Design

This was a longitudinal observational study involving pediatric patients who received cochlear implants at Dr. Guillermo Grant Benavente Regional Clinical Hospital in Concepción, Chile, between August 2013 and December 2019. The sampling was non-randomized and based on convenience, limiting the sample to those who met the clinical and procedural inclusion criteria. Of the 64 children who received cochlear implants during this period through public health funding, 57 provided informed consent to participate. However, only 18 met the full eligibility criteria for this study.

The discrepancy between the initial 57 and the final 18 participants was primarily due to the following reasons: failure to meet specific eligibility criteria (e.g., absence of associated disabilities or incomplete audiological data), inconsistent use of the cochlear implant, and missing follow-up assessments 12 months post-activation. No control group was included in this study due to its descriptive nature and focus on a historically underrepresented population.

### 2.2. Eligibility Criteria

Participants included in the final sample had to meet the following criteria:

- A confirmed diagnosis of severe or profound bilateral sensorineural hearing loss.
- At least one additional disability affecting neurodevelopment and/or a syndromic diagnosis associated with HL.
- Anatomically intact auditory nerves and a cochlea without malformations, confirmed via imaging.
- Receipt of a unilateral cochlear implant through the public health system.
- Completion of auditory speech perception assessments both pre-implantation and 12 months post-activation.
- Consistent use of the cochlear implant sound processor.
- No postsurgical complications requiring reimplantation.

### 2.3. Demographics

The final sample consisted of 18 children (6 girls and 12 boys), aged between 2.0 and 16.1 years at the time of cochlear implant activation. Additional diagnostic details are provided in Table 2.

### 2.4. Speech Perception Testing Procedures

Auditory speech perception was evaluated using speech perception tools recommended by the Chilean Ministry of Health. These included assessments of suprasegmental perception, vowel and consonant recognition, and sentence discrimination tasks. Based on the combined results, each participant was assigned a corresponding CAP category. All testing procedures were conducted by a certified speech and language pathologist at the hospital.

The assessments were carried out during routine follow-up sessions, each lasting between 45 min and 1 h, in a designated speech therapy room. Although the environment was quiet, it was not acoustically treated. Auditory stimuli were presented verbally by the speech–language therapist; however, the intensity of the stimuli was not quantified using a sound level meter. Pre-implantation assessments were conducted within 1–2 months prior to cochlear implant surgery, while post-implantation evaluations were performed 12 months after device activation, as part of the routine follow-up protocol.

### 2.5. Statistical Analysis

Descriptive statistics were used to summarize demographic and clinical data. The distribution of key variables was assessed using the Shapiro–Wilk test, and due to non-normality, non-parametric methods (Wilcoxon signed-rank test) were used to compare CAP scores pre- and post-implantation.

All statistical analyses were conducted using Jamovi software (version 2.3.28). These methods were chosen based on the nature of the data (ordinal and small sample size) and were supported by recommendations from previous studies using CAP scales in similar populations.

## 3. Results

The final sample consisted of 18 children with a median age of cochlear implant activation of 3.10 years (range: 2.0–16.1 years). The mean age at hearing loss detection was 1.6 years (median = 0.9 years), although data for this variable were missing in two cases. Table 2 summarizes the types and frequencies of additional disabilities present in the study population.

At baseline (pre-implantation), most participants scored 0 on the CAP scale, indicating no awareness of sound. Twelve months after cochlear implant activation, the median CAP score increased to 2. The range of improvements observed was from 0 to 2 CAP levels, with a median improvement of 1.

Table 3 summarizes these descriptive statistics and sample distribution. The 201 standard deviations indicate a high degree of dispersion in the age at detection, and 202 indicates the age at implant activation, reflecting a wide range of values.

Given the small sample size and non-normal distribution of the data (confirmed by the Shapiro–Wilk test, *p* < 0.05), the Wilcoxon signed-rank test was used to compare auditory perception scores before and after implantation. The analysis revealed a statistically significant improvement in CAP score post-implantation (*p* < 0.001) as shown in Table 4.

**Figure 1:**
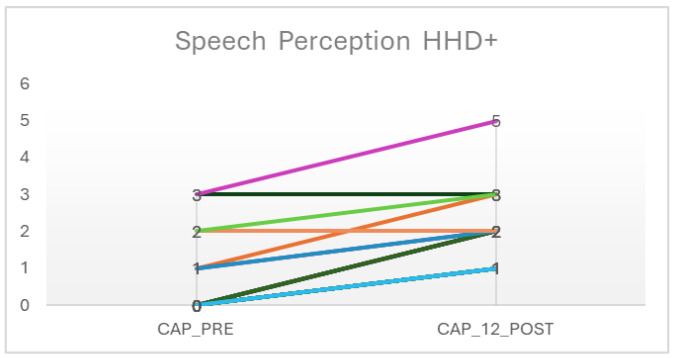
Pre-/post-IC speech perception.

Figure 1 illustrates the distribution of CAP scores before and after cochlear implantation. While improvements were observed among most participants, two children showed no change in auditory perception over the 12-month period.

**Figure 2:**
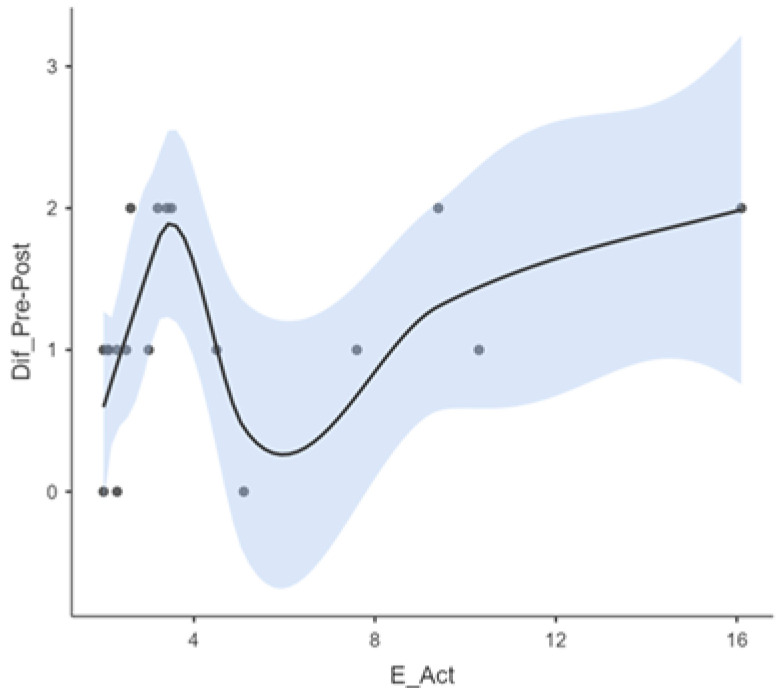
Progress according to activation age.

Figure 2 illustrates a smoothed regression curve depicting the relationship between age at cochlear implant activation and improvement in auditory performance. A non-linear trend was observed: children implanted before the age of four demonstrated a steeper and more rapid trajectory of improvement. This was followed by a deceleration in progress between ages four and eight, and a modest upward trend thereafter. These patterns underscore the nuanced influence of age at implantation on auditory outcomes, suggesting that the timing of activation may differentially impact auditory development across age groups.

The results indicate that the relationship between age at activation and improvement in auditory speech perception is both complex and non-linear. The data suggest that the effect of activation age on auditory gains varies across different developmental stages. This may account for the presence of inflection points, where age activation appears to exert a more pronounced influence on the degree of improvement observed.

## 4. Discussion

The results of this study support the existing literature indicating that cochlear implantation provides measurable benefits in auditory perception for children with hearing loss and additional disabilities [6,8,14,22]. After 12 months of device activation, most children in the sample demonstrated progression on the CAP scale, with a median improvement from 0 to 2. Although many remain in the lower Categories Of Auditory Performance, even modest gains in this population are clinically meaningful given the complex neurodevelopmental challenges they face [5,23].

One of the most compelling findings was the non-linear relationship observed between age at activation and auditory progress. While children implanted before the age of 4 generally showed faster and more consistent improvement [21,24], the data revealed a second upward trend in progress among children implanted after age 8 [25]. This pattern may reflect underlying neurodevelopmental heterogeneity or the influence of cumulative auditory deprivation [26]. Although this trend has been alluded to in past studies, our data highlights its significance in children with complex conditions and warrant further investigation through modeling approaches like generalized linear mixed-effects models.

Moreover, the interplay between specific disabilities and auditory progress emerged as a potentially important area of study. While our sample was not large enough to perform subgroup analyses, anecdotal observations suggest that children with global developmental delay or visual impairments may respond differently than those with ASD or severe motor impairments. For example, children with ASD showed more variability in CAP progression, possibly due to attentional or behavioral factors. These trends merit further investigation into larger, stratified samples [4,5,6,27].

Another novel contribution of this study is its contextual focus on a publicly funded cochlear implant program in southern Chile, which has only recently begun including children with additional disabilities. The results emphasize the value of expanding eligibility criteria to include more diverse populations, as minimal auditory gains can enhance quality of life and support nonverbal communication strategies in children with limited language acquisition potential [5,6,8].

Although the findings align with previous reports that emphasize early implantation as a key predictor of success [9,28,29], our data underscore that timing alone is insufficient [5,6,8,15]. Individual developmental profiles, access to consistent auditory training, and family involvement play equally important roles in auditory progress. These factors should be considered in future studies and in clinical practice when counseling families.

Despite its contributions, the study has several limitations. The small sample size restricts the statistical power of our analysis and limits the generalizability of findings [6,14]. The absence of a control group precludes comparisons with children without additional disabilities, making it difficult to isolate the impact of comorbidities on cochlear implant outcomes. Additionally, while the CAP scale is a valid and widely used tool to assess auditory performance [18,19], it may not capture subtle auditory behaviors or progress in preverbal children. The integration of additional outcome measures, such as the IT-MAIS [30] or LittlEARS [31], would provide a more comprehensive evaluation of auditory development.

Given these considerations, the results presented here support the value of cochlear implantation in children with hearing loss and additional disabilities, even when progress is modest by conventional standards. Achieving CAP categories 2 or 3 within the first year—equivalent to responding to speech and recognizing environmental sounds—represents a substantial improvement in auditory access. For children with severe neurodevelopmental challenges, this access may enhance environmental interaction, support nonverbal communication strategies, and contribute meaningfully to overall development [4,5,32,33]. However, expectations regarding cochlear implant outcomes in this population must be tailored to their individual developmental profiles. A strictly linguistic objective may not always be appropriate. To capture the broader impact of auditory rehabilitation—on quality of life, autonomy, and social participation—future research should incorporate multidimensional assessments and family-centered outcome measures [7,14,32,34].

Future research should adopt more robust methodological approaches. Specifically, the inclusion of a control group—such as children with cochlear implants but without additional disabilities—would strengthen causal inferences. Studies should also employ advanced statistical methods, such as generalized linear mixed-effects models, to better model the effects of age at implantation, type of disability, training intensity, and family involvement.

Beyond auditory perception, there is a growing need to evaluate broader and more comprehensive outcome measures, including language development, cognitive function, and quality of life. Multidimensional assessment tools and long-term follow-up beyond 12 months will provide a more complete picture of how cochlear implantation contributes to the developmental trajectory of these children.

Lastly, future studies should examine the role of family support, early intervention services, and educational inclusion as mediating factors in auditory and language outcomes. A family-centered, interdisciplinary approach remains essential to optimizing success in this diverse and underrepresented population.

## 5. Conclusions

This study highlights the positive impact of cochlear implantation on auditory perception in children with hearing loss and additional disabilities, even when improvements appear modest on traditional scales. Although many participants remained in lower CAP categories after 12 months, their increased awareness of environmental and speech-related sounds represents a meaningful step toward greater communication and interaction with their environment.

These findings support the inclusion of children with complex developmental profiles in cochlear implant programs, provided that expectations are tailored and support systems are in place. Early implantation, consistent use of the device, and individualized auditory rehabilitation appear to be key factors in promoting auditory progress in this diverse population.

## Figures and Tables

**Table 1:** Category of Auditory Performance.

0	No awareness of environmental sounds
1	Awareness of environmental sounds
2	Response to speech sounds
3	Identification of environmental sounds
4	Discrimination of some speech sounds without lip reading
5	Comprehension of common sentences without lip reading
6	Conversation comprehension without lip reading
7	Use of the telephone with a known speaker

Ref. [18].

**Table 2:** Additional disabilities.

Additional Disabilities	Frequency	% of Total	% Accumulated
Global developmental delay	2	11.1%	11.1%
Autism spectrum disorder, cleft of soft and hard palates	1	5.6%	16.7%
Waardenburg syndrome	1	5.6%	22.2%
Autism spectrum disorder	1	5.6%	27.8%
Prematurity, microcephaly, hypothyroidism	1	5.6%	33.3%
Cerebral palsy (spastic tetraparesis), prematurity, hydrocephalus, global developmental delay, epilepsy	1	5.6%	38.9%
Waardenburg syndrome, mild intellectual disability	1	5.6%	44.4%
Global developmental delay, hypotony, autism spectrum disorder	1	5.6%	50.0%
Prematurity, cytomegalovirus, retinitis	1	5.6%	55.6%
Prematurity, global developmental delay, learning disability	1	5.6%	61.1%
Severe spinal cord aplasia, learning disability	1	5.6%	66.7%
Global developmental delay, learning disability	1	5.6%	72.2%
Extrapyramidal cerebral palsy, global developmental delay, microcephaly, prematurity	1	5.6%	77.8%
Prematurity, mixed cerebral palsy	1	5.6%	83.3%
Prematurity, global developmental delay, strabismus	1	5.6%	88.9%
Prematurity, severe hemolytic disease due to Rh- mother, retinopathy	1	5.6%	94.4%
Usher syndrome, corneal dystrophy	1	5.6%	100.0%

**Table 3:** Descriptive analysis of the sample.

	A_DETEC	A_Act	CAP_Preimp	CAP_Post	Dif_Pre-Post
N	16	18	18	18	18
Lost	2	0	0	0	0
Average	1.61	4.67	0.722	1.94	1.17
Median	0.950	3.10	0.00	2.00	1.00
Standard Deviation	1.53	3.83	1.07	1.06	0.707
Minimum	0.110	2.00	0	1	0
Maximum	5.00	16.1	3	5	2
Shapiro–Wilk W	0.782	0.721	0.706	0.796	0.807
Shapiro–Wilk *p*-value	0.002	<0.001	<0.001	0.001	0.002

A_DETEC: age detection HL/D; A_Act: age of activation CI; CAP_Preimp: CAP pre-implantation; CAP_Post: CAP post-implantation; Dif_Pre-Post: difference between pre- and post-implantation.

**Table 4:** Wilcoxon signed-rank test.

			Statistic	*p*	Mean Difference	EE of the Difference		Effect Size
CAP_Preimp	CAP_Post	W of Wilcoxon	0.00 ^a^	<0.001	−1.50	0.152	Biserial correlation of ranges	−1.00

Note: H_a_ µMeasure 1 − Measure 2 ≠ 0. ^a^ Two pairs of values were repeated.

## Data Availability

The data sets presented in this article are not available because the data are part of an ongoing Ph.D. thesis.

## Abbreviations

The following abbreviations are used in this manuscript:

ASD	Autism spectrum disorder
CAP	Category of Auditory Performance
CI	Cochlear implant
HL/D	Hearing loss or deafness

## References

[1] World Health Organization (2021). World Report on Hearing.

[2] Yoshinaga-Itano C., Manchaiah V., Hunnicutt C. (2021). Outcomes of Universal Newborn Screening Programs: Systematic Review. J. Clin. Med..

[3] Edmond K., Chadha S., Hunnicutt C., Strobel N., Manchaiah V., Yoshinga-Itano C. (2022). Effectiveness of universal newborn hearing screening: A systematic review and meta-analysis. J. Glob. Health.

[4] Joint Committee on Infant Hearing (2019). Year 2019 JCIH Position Statement.pdf. https://digitalcommons.usu.edu/jehdi/vol4/iss2/1/.

[5] Núñez-Batalla F., Jáudenes-Casaubón C., Sequí-Canet J.M., Vivanco-Allende A., Zubicaray-Ugarteche J. (2021). Sordera infantil con discapacidad asociada (DA+): Recomendaciones CODEPEH. Acta Otorrinolaringol. Esp..

[6] Wakil N., Fitzpatrick E.M., Olds J., Schramm D., Whittingham J. (2014). Long-term outcome after cochlear implantation in children with additional developmental disabilities. Int. J. Audiol..

[7] Hayward D.V., Ritter K., Grueber J., Howarth T. (2013). Outcomes That Matter for Children with Severe Multiple Disabilities who use Cochlear Implants: The First Step in an Instrument Development Process. Can. J. Speech Lang. Pathol. Audiol..

[8] Cejas I., Hoffman M., Quittner A. (2015). Outcomes and benefits of pediatric cochlear implantation in children with additional disabilities: A review and report of family influences on outcomes. Pediatr. Health Med. Ther..

[9] Ching T.Y., Dillon H., Marnane V., Hou S., Day J., Seeto M., Crowe K., Street L., Thomson J., Van Buynder P. (2013). Outcomes of Early- and Late-Identified Children at 3 Years of Age: Findings From a Prospective Population-Based Study. Ear Hear..

[10] Ministerio de Salud (2013). Guía Clínica Auge Tratamiento de la Hipoacusia Moderada en Menores de 2 Años.

[11] Ministerio de Salud (2008). Guía de Práctica Clínica Implante Coclear. Rehabilitación de Personas en Situación de Discapacidad por Hipoacusia Sensorioneural Severa a Profunda Bilateral.

[12] Ministerio de Salud (2009). Hipoacusia Neurosensorial Bilateral del Prematutro.

[13] (2018). Minsal. Resumen Ejecutivo. Guía de Práctica Clínica Hipoacusia en Recién Nacidos, Niños y Niñas Menores de 4 Años. https://docs.bvsalud.org/biblioref/2024/02/967241/hipoacusia-neurosensorial-bilateral-del-prematuro-guias-clinica_o6oqB6h.pdf.

[14] Rawes C., Ngaage L.M., Mackenzie R., Martin J., Cordingley A., Raine C. (2021). A review of the outcomes of children with designated additional needs receiving cochlear implantation for severe to profound hearing loss. Cochlear Implant. Int..

[15] Wiley S., John R.S., Lindow-Davies C. (2022). Children Who Are Deaf or Hard of Hearing PLUS. https://www.infanthearing.org/ehdi-ebook/.

[16] Chapman D.A., Stampfel C.C., Bodurtha J.N., Dodson K.M., Pandya A., Lynch K.B., Kirby R.S. (2011). Impact of Co-Occurring Birth Defects on the Timing of Newborn Hearing Screening and Diagnosis. Am. J. Audiol..

[17] Mortari Moret A., RehAB Latin American Leaders Committee (2023). Pruebas y Protocolos de Evaluación en Español y Portugués. La Práctica Auditivo Verbal (PAV) en Latinoamérica. Intervención en la Población Infantil con Pérdida Auditiva y sus Familias.

[18] Archbold S., Lutman M.E., Nikolopoulos T. (1995). Categories of Auditory Performance. Ann. Otol. Rhinol. Laryngol..

[19] Albalawi Y., Nidami M., Almohawas F., Hagr A., Garadat S.N. (2019). Categories of Auditory Performance and Speech Intelligibility Ratings in Prelingually Deaf Children with Bilateral Implantation. Am. J. Audiol..

[20] Archbold S., Nikolopoulos T.P., O’Donoghue G.M., Lutman M.E. (1998). Educational placement of deaf children following cochlear implantation. Br. J. Audiol..

[21] Jalil-Abkenar S.S., Ashori M., Pourmohamadreza-Tajrishi M., Hasanzadeh S. (2013). Auditory Perception and Verbal Intelligibility in Children with Cochlear Implant, Hearing Aids and Normal Hearing. Pract. Clin. Psychol..

[22] Rafferty A., Martin J., Strachan D., Raine C. (2013). Cochlear implantation in children with complex needs—Outcomes. Cochlear Implants Int..

[23] Davidson L.S. (2020). La Audición Acústica Temprana y el Papel de la Percepción Segmental y Suprasegmental del Habla en el Lenguaje Hablado y la Alfabetización.

[24] Van de Velde D.J., Schiller N.O., Levelt C.C., Van Heuven V.J., Beers M., Briaire J.J., Frijns J.H. (2019). Prosody perception and production by children with cochlear implants. J. Child Lang..

[25] Magalhães A., Samuel P., Goffi-Gomez M., Tsuji R., Brito R., Bento R. (2014). Audiological outcomes of cochlear implantation in Waardenburg Syndrome. Int. Arch. Otorhinolaryngol..

[26] Kral A., Sharma A. (2012). Developmental neuroplasticity after cochlear implantation. Trends Neurosci..

[27] Lachowska M., Pastuszka A., Łukaszewicz-Moszyńska Z., Mikołajewska L., Niemczyk K. (2018). Cochlear implantation in autistic children with profound sensorineural hearing loss. Braz. J. Otorhinolaryngol..

[28] Ching T.Y.C.C., Dillon H., Leigh G., Cupples L. (2018). Learning from the Longitudinal Outcomes of Children with Hearing Impairment (LOCHI) study: Summary of 5-year findings and implications. Int. J. Audiol..

[29] Dettman S.J., Dowell R.C., Choo D., Arnott W., Abrahams Y., Davis A., Dornan D., Leigh J., Constantinescu G., Cowan R. (2016). Long-term Communication Outcomes for Children Receiving Cochlear Implants Younger Than 12 Months. Otol. Neurotol..

[30] Zimmerman-Phillips S., Robbins A.M., Osberger M.J. (2000). Assessing Cochlear Implant Benefit in Very Young Children. Ann. Otol. Rhinol. Laryngol..

[31] Obrycka A., Lorens A., García J.L.P., Piotrowska A., Skarzynski H. (2017). Validation of the LittlEARS Auditory Questionnaire in cochlear implanted infants and toddlers. Int. J. Pediatr. Otorhinolaryngol..

[32] McDonnell S. (2014). The Ling Sound Test: What Is Its Relevance in the New Zealand Classroom?. Kairaranga.

[33] Jatana K.R., Thomas D., Weber L., Mets M.B., Silverman J.B., Young N.M. (2013). Usher Syndrome: Characteristics and Outcomes of Pediatric Cochlear Implant Recipients. Otol. Neurotol..

[34] Young N.M., Weil C., Tournis E. (2016). Redefining Cochlear Implant Benefits to Appropriately Include Children with Additional Disabilities. Pediatric Cochlear Implantation.

